# Tuning the Absorption Spectrum of Polydopamine via Post-Synthetic Oxidation with Bobbit’s Salt

**DOI:** 10.3390/molecules31101664

**Published:** 2026-05-14

**Authors:** Cheng Chang, Yiming Yin, Sheng Long, Defa Hou, Fulin Yang, Xu Lin, Yunwu Zheng, Yuan Zou

**Affiliations:** 1 National Joint Engineering Research Center for Highly-Efficient Utilization Technology of Forestry Resources, Southwest Forestry University, Kunming 650224, China; changc@swfu.edu.cn (C.C.); y18656096337@163.com (Y.Y.); ls0901@swfu.edu.cn (S.L.); houdefa001@163.com (D.H.); yangfulin0309@163.com (F.Y.); linxunefu@126.com (X.L.); zyw85114@163.com (Y.Z.); 2 State Key Laboratory of Advanced Polymer Materials, Sichuan University, Chengdu 610065, China

**Keywords:** polydopamine, Bobbit’s salt, absorption spectrum, oxidation

## Abstract

Polydopamine (PDA) is a promising biomimetic material, but its structural complexity hinders rational control over its light absorption properties. The purpose of this study was to develop a simple post-synthetic method to tune the absorption spectrum of PDA using Bobbit’s salt (4-acetylamino-2,2,6,6-tetramethylpiperidine-1-oxoammonium salt) as a mild oxidant. Conventional PDA nanoparticles were treated with Bobbit’s salt either in pure water or in a 1:1 methanol–water mixture to obtain two modified samples. Structural analysis conducted using Fourier transform infrared spectroscopy, X-ray photoelectron spectroscopy, and mass spectrometry demonstrated that Bobbit’s salt selectively oxidized catechol units to ortho-benzoquinone moieties, with the C–O/C=O ratio decreasing from 71:29 in the untreated PDA to 51:49 in the water-treated sample, while nitrogen functionalities remained unchanged. Consequently, the sample prepared in pure water showed generally lower absorbance across the visible–near-infrared range, whereas the sample prepared in the methanol–water mixture exhibited enhanced ultraviolet absorption but reduced near-infrared absorption. When coated onto polyvinylidene fluoride membranes, the water-treated PDA produced a brighter and more reddish-yellow appearance. On transparent poly(methyl methacrylate) substrates, the same coating also enhanced ultraviolet blocking and reduced visible transmittance. These findings conclude that Bobbit’s salt is an effective and selective reagent for tailoring the optical properties of PDA, with potential applications in protective coatings and light-modulating materials.

## 1. Introduction

Polydopamine (PDA) has emerged as one of the most versatile bio-inspired materials in recent decades [1,2,3,4,5,6]. Derived from the oxidative polymerization of dopamine under alkaline conditions, PDA inherits many features of natural melanins, including broad ultraviolet–visible–near-infrared (UV-vis-NIR) absorption, free-radical scavenging, metal ion chelation, and excellent adhesion to virtually all types of surfaces [7,8,9,10]. These unique properties have enabled applications in surface functionalization, drug delivery, photothermal therapy, environmental remediation, and energy conversion [11,12,13,14]. A particularly attractive characteristic of PDA is its intrinsic photothermal effect, which stems from the efficient non-radiative decay of excitons generated upon light absorption [15]. However, the same structural heterogeneity that gives rise to broadband absorption also makes it difficult to rationally tune the optical response of PDA. The polymer network is composed of a complex mixture of non-cyclized dopamine units, 5,6-dihydroxyindole (DHI), indole-5,6-quinone (IQ), and their oligomers, held together by covalent bonds and non-covalent interactions such as π-π stacking and cation-π forces [16,17,18,19]. Consequently, controlling the absorption spectrum of PDA remains a significant challenge.

Numerous approaches have been explored to modify the optical properties of PDA. Copolymerization of dopamine with other monomers, such as aniline or pyrrole, can introduce additional conjugated segments and alter the absorption profile [20,21]. Doping with metal ions (e.g., Fe^3+^ and Cu^2+^) or incorporating noble metal nanoparticles has been shown to enhance NIR absorption through localized surface plasmon resonance or charge-transfer interactions [13,22,23,24]. Post-synthetic chemical modifications, including reduction or oxidation, have also been investigated [25,26]. Among these, the construction of donor–acceptor (D-A) pairs within the PDA structure has proven particularly effective for narrowing the bandgap and increasing electron delocalization, thereby improving light harvesting across a broad spectral range [14,15]. For instance, Zou et al. demonstrated that copolymerizing dopamine with 2,2,6,6-tetramethylpiperidine-1-oxyl (TEMPO) generates D-A structures that significantly enhance the photothermal efficiency of PDA nanoparticles [14]. Nevertheless, most of these strategies require careful control of the polymerization conditions or the use of specialized co-monomers. A simple, post-synthetic method that selectively oxidizes specific functional groups in pre-formed PDA while preserving the overall particle integrity would be highly desirable.

**Scheme 1:**
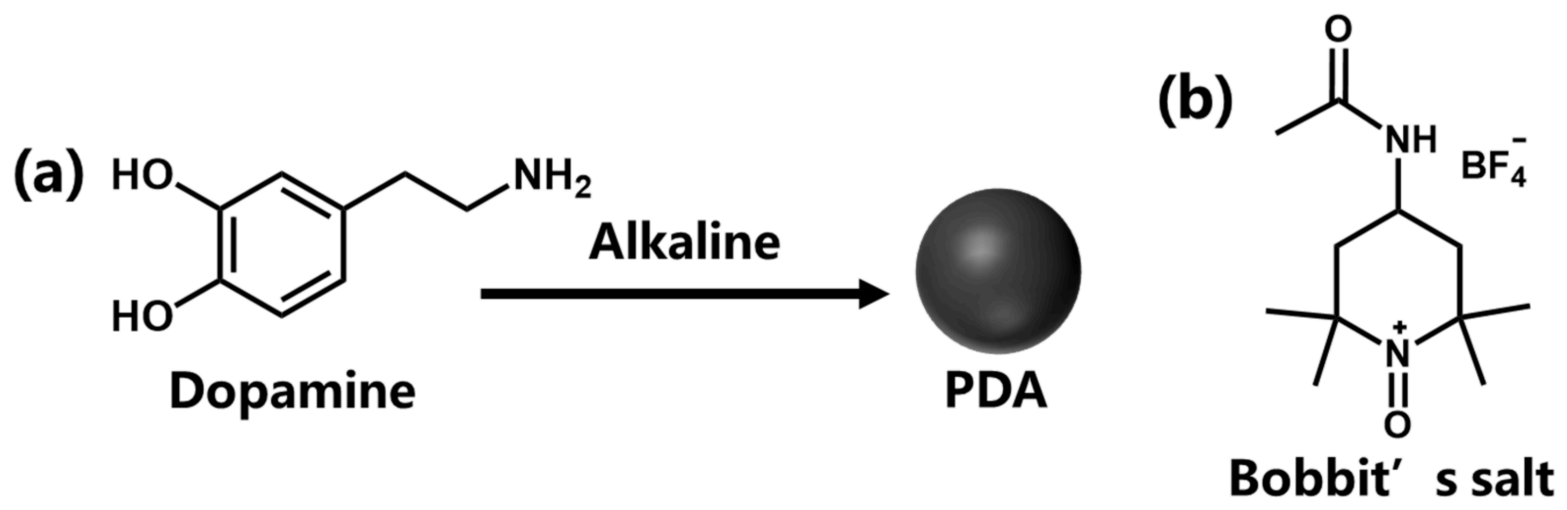
(**a**) Synthesis of PDA through polymerization of dopamine in alkaline condition. (**b**) Chemical structure of Bobbit’s salt.

In this work, we report a mild and effective post-synthetic treatment of PDA using Bobbit’s salt (4-acetylamino-2,2,6,6-tetramethylpiperidine-1-oxoammonium salt), a well-known oxoammonium oxidant widely used in organic synthesis for the selective oxidation of alcohols and phenols to carbonyl compounds [27,28,29]. Unlike TEMPO, which is typically introduced during polymerization, Bobbit’s salt can be applied after PDA formation (Scheme 1). We show that immersing conventional PDA nanoparticles (PDA-0) in Bobbit’s salt solution, either in pure water or in a 1:1 methanol–water mixture, leads to selective oxidation of catechol groups to ortho-benzoquinone moieties without affecting the nitrogen-containing units. This transformation is evidenced by Fourier transform infrared spectroscopy, X-ray photoelectron spectroscopy, and mass spectrometry. The modified samples, denoted PDA-1 and PDA-2, exhibit distinct absorption spectral changes: PDA-1 shows generally lower absorbance across the visible-NIR range, while PDA-2 displays enhanced UV absorption but reduced NIR absorption. Colorimetric analysis of PDA-coated PVDF membranes reveals a brighter, more reddish-yellow appearance after treatment, and transmittance measurements on substrates demonstrate improved UV-blocking and visible-light shielding. Collectively, our findings establish Bobbit’s salt as a convenient, selective, and scalable reagent for post-synthetic tuning of the optical properties of polydopamine. This approach opens new avenues for designing PDA-based materials for protective coatings, optical filters, solar-modulating layers, and other light-management applications.

## 2. Results and Discussion

### 2.1. Synthesis and Morphological Characterization

Polydopamine nanoparticles were first prepared by dissolving dopamine hydrochloride in a water–methanol mixed solvent followed by the addition of ammonia to adjust the pH to alkaline. The resulting conventional polydopamine sample was designated as PDA-0. To modify the absorption properties, PDA-0 was treated with Bobbit’s salt at a mass ratio of 1:8. Two different treatment conditions were applied: one in aqueous Bobbit’s salt solution yielding PDA-1, and the other in a 1:1 methanol–water mixture containing Bobbit’s salt yielding PDA-2. After the treatment, the nanoparticles were collected by centrifugation and washed with water.

**Figure 1:**
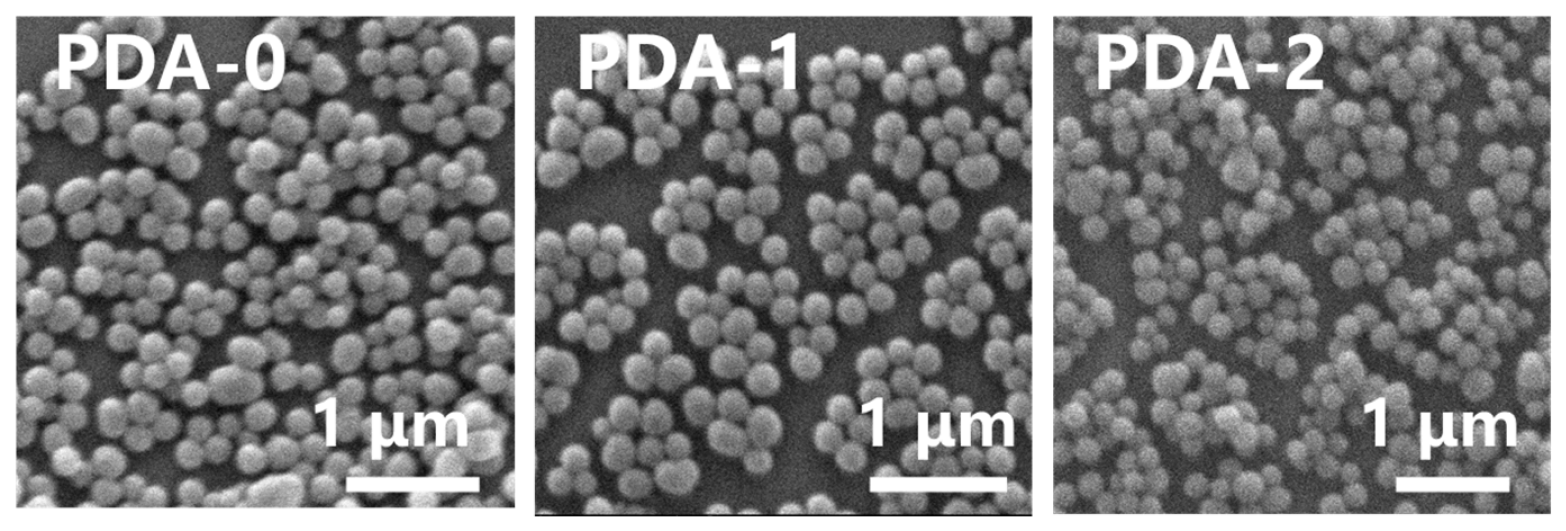
SEM images of PDA-0, PDA-1, and PDA-2.

Scanning electron microscopy images of PDA-0, PDA-1, and PDA-2 are shown in Figure 1. All three samples exhibited spherical or quasi spherical particle morphologies with relatively uniform size distributions. The average particle diameters were measured as 201 ± 25 nm for PDA-0, 207 ± 28 nm for PDA-1, and 204 ± 29 nm for PDA-2. These values indicate that the Bobbit’s salt treatment did not significantly alter the particle size or the size distribution regardless of the solvent used. Therefore, any changes in optical properties observed later can be attributed to chemical structural modifications rather than to morphological or size effects.

### 2.2. Ultraviolet–Visible–Near-Infrared Absorption

**Figure 2:**
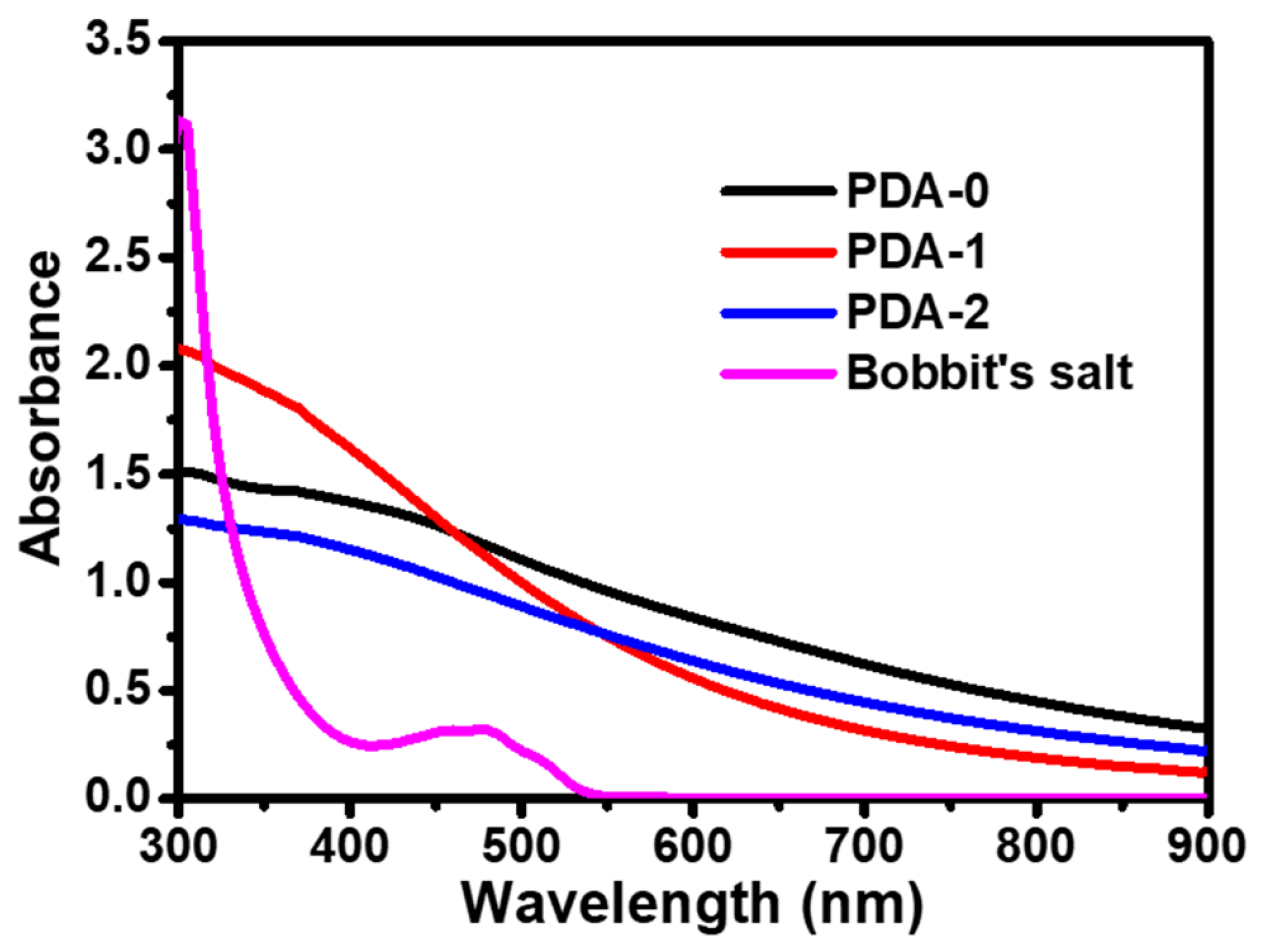
UV-vis spectra of PDA-0, PDA-1, PDA-2, and Bobbit’s salt.

The ultraviolet–visible–near-infrared absorption spectra of PDA-0, PDA-1, PDA-2 and the pristine Bobbit’s salt are presented in Figure 2. All polydopamine samples exhibited the characteristic broadband absorption with a gradual decrease in absorbance as the wavelength increased from 300 to 900 nm. Compared with conventional PDA-0, the sample prepared in aqueous Bobbit’s salt solution (PDA-1) showed higher absorbance in the range of 300–450 nm, while its absorbance decreased noticeably in the visible and near-infrared region beyond 450 nm. In contrast, the sample prepared in the water–methanol mixed solvent (PDA-2) exhibited a spectral shape nearly identical to that of PDA-0 but with a generally lower absorbance across the entire measured range. For reference, Bobbit’s salt alone showed absorption mainly below 400 nm and negligible absorbance at longer wavelengths. These spectral variations suggest that the interaction between Bobbit’s salt and polydopamine depends strongly on the solvent composition, leading to different degrees of structural transformation.

### 2.3. Fourier Transform Infrared Spectroscopy

**Figure 3:**
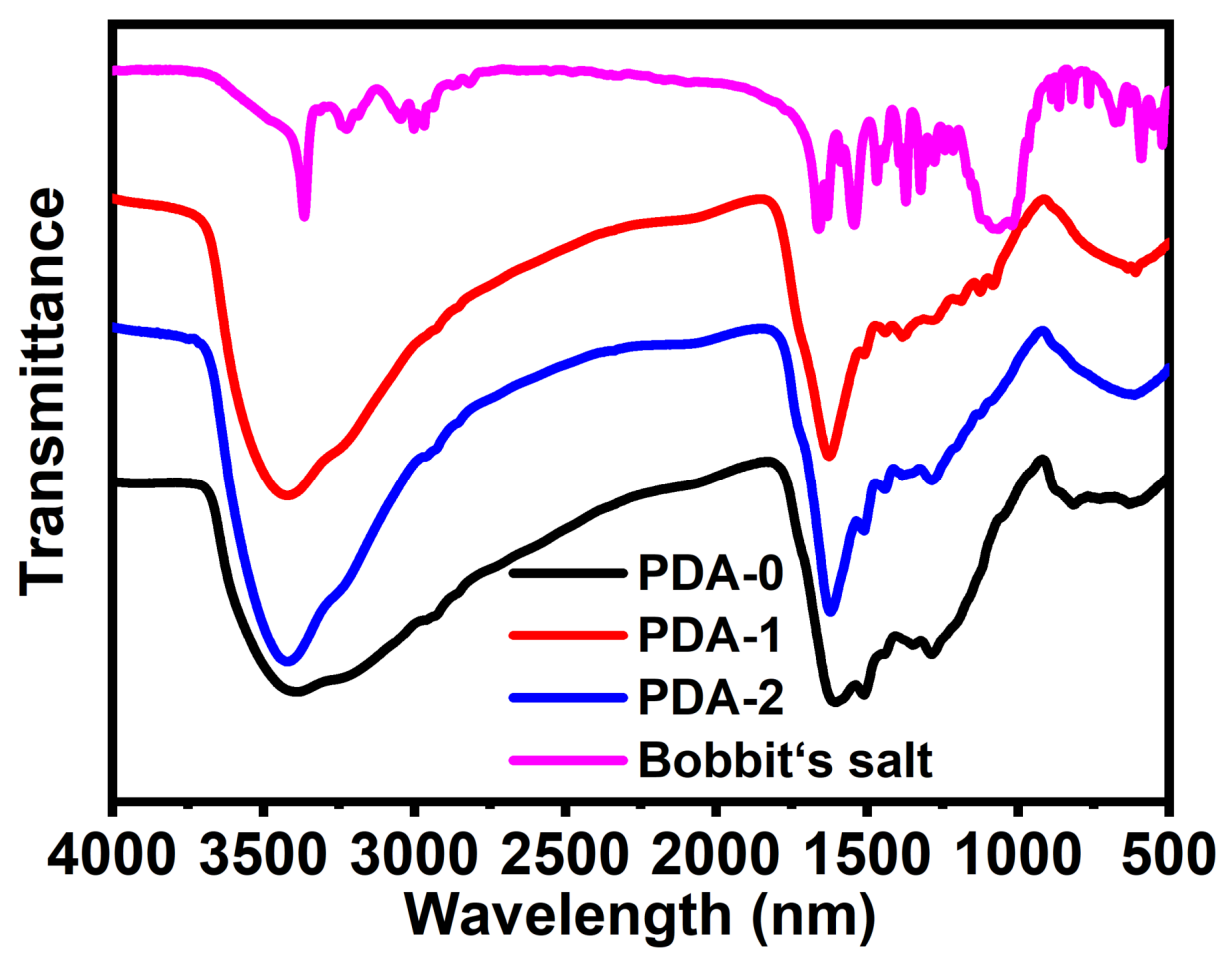
FTIR spectra of PDA-0, PDA-1, PDA-2, and Bobbit’s salt.

Fourier transform infrared spectroscopy was used to investigate the chemical changes in the polydopamine structure after Bobbit’s salt treatment, as shown in Figure 3. The spectrum of PDA-0 exhibited strong characteristic peaks at approximately 3500 cm^−1^ corresponding to phenolic hydroxyl groups and at around 1500 cm^−1^ assigned to aromatic ring vibrations [30]. A clear carbonyl absorption near 1650 cm^−1^ was also observed. This carbonyl peak did not decrease for PDA-1 and PDA-2; instead, it increased relative to PDA-0, which is consistent with the formation of quinone structures. The broad signals in the 2000–3750 cm^−1^ region are now attributed to O–H stretching envelopes together with their overtones; the observed narrowing and contraction of these bands correlate with the reduced hydroxyl content after oxidation. Specifically, the phenolic O–H band at ∼3500 cm^−1^ and the aromatic C=C band at ∼1500 cm^−1^ decrease progressively from PDA-0 to PDA-1 to PDA-2, indicating a marked reduction in the content of phenolic hydroxyl groups and aromatic rings. The most pronounced decrease occurred in PDA-2, indicating a marked reduction in the content of phenolic hydroxyl groups and aromatic rings. This observation is consistent with the conversion of these structural units into benzoquinone moieties. Furthermore, the absorption band shifts from 1598 cm^−1^ in PDA-0 to 1625 cm^−1^ in PDA-2 shifted to a higher wavenumber, which further supports the transformation toward a more quinone-rich structure [31].

Taken together, the spectral and structural analyses demonstrate that Bobbit’s salt can effectively modify the chemical composition of polydopamine. The extent of modification is influenced by the solvent used during treatment. The water–methanol mixture promotes a more profound conversion of phenolic groups to quinone structures, which in turn alters the absorption spectrum, particularly by enhancing ultraviolet absorption while suppressing near-infrared absorption. These results provide a basis for tuning the optical properties of polydopamine-based materials through a simple post-synthetic treatment with Bobbit’s salt.

### 2.4. X-Ray Photoelectron Spectroscopy

**Figure 4:**
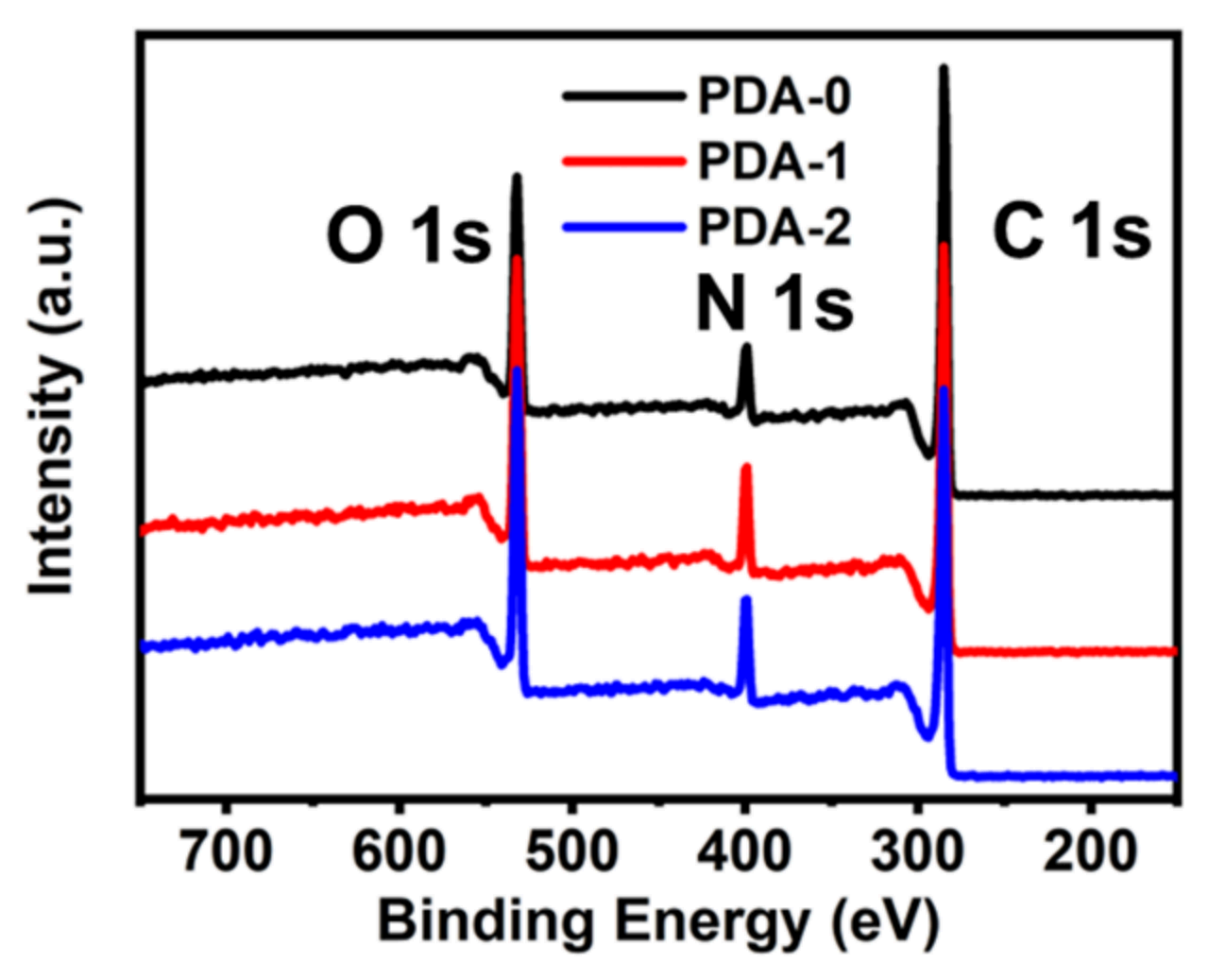
XPS survey spectra of PDA-0, PDA-1, and PDA-2.

**Figure 5:**
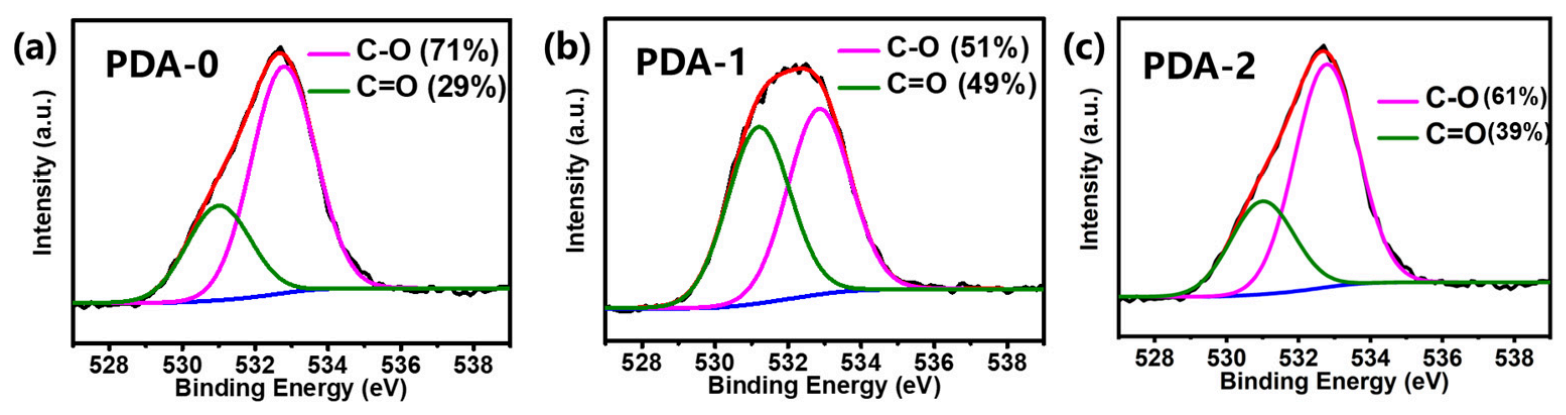
O 1s peaks in XPS spectra of (**a**) PDA-0, (**b**) PDA-1, and (**c**) PDA-2. The black line represents the raw experimental data; the red line is the total fitted curve; the blue line is the background baseline; pink (C–O) and green (C=O) lines are the deconvoluted components.

X-ray photoelectron spectroscopy was performed to further elucidate the chemical changes induced by Bobbit’s salt treatment. Survey spectra of PDA-0, PDA-1 and PDA-2 confirmed the presence of carbon, oxygen and nitrogen in all the samples (Figure 4). High-resolution O 1s and N 1s spectra were deconvoluted to quantify the different chemical states. In the O 1s spectrum of PDA-0, the C O bond contribution was 71% with a typical binding energy around 533.0 eV, while the C=O bond accounted for 29% at approximately 531.5 eV (Figure 5a) [32]. After treatment with Bobbit’s salt, PDA-1 showed a significant decrease in C–O bonds to 51% and a corresponding increase in C=O bonds to 49% (Figure 5b) [33]. For PDA-2, which was treated in the water–methanol mixture, the O 1s spectrum gave a C–O contribution of 61% and a C=O contribution of 39% (Figure 5c). This indicates that the presence of methanol moderates the oxidation reaction, leading to a lower degree of quinone formation compared to PDA-1.

**Figure 6:**
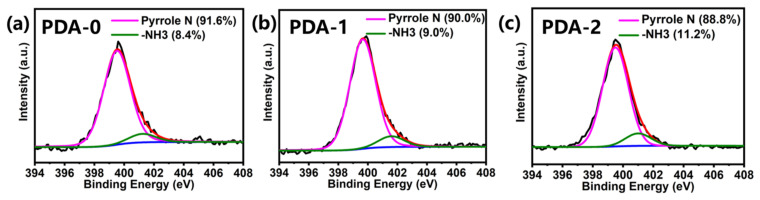
N 1s peaks in XPS spectra of (**a**) PDA-0, (**b**) PDA-1, and (**c**) PDA-2. The black line represents the raw experimental data; the red line is the total fitted curve; the blue line is the background baseline; pink (pyrrole N) and green (-NH_3_) lines are the deconvoluted components.

The N 1s spectra of PDA-0 and PDA-1, however, revealed only minor differences. In PDA-0, the pyrrolic nitrogen (around 399.8 eV) contributed 91.6% and the primary amine NH_2_ (around 401.2 eV) contributed 8.4% (Figure 6a) [34]. For PDA-1, these values were 90.0% and 9.0%, respectively (Figure 6b). For PDA-2, the pyrrolic nitrogen contributed 88.8% and the NH_2_ contributed 11.2% (Figure 6c). The nearly unchanged nitrogen chemical environment suggests that the oxidation induced by Bobbit’s salt primarily targets the catechol groups rather than the nitrogen-containing units. Therefore, the electronic structure of the polydopamine backbone remains largely intact while the quinone content is increased.

The XPS results show that the oxidation extent follows the order PDA-1 (C=O 49%) > PDA-2 (C=O 39%) > PDA-0 (C=O 29%). This indicates that pure water is a more effective medium for Bobbit’s salt to oxidize polydopamine than the water–methanol mixture. A plausible explanation is that methanol, a known radical scavenger, may partially quench the reactive nitroxide radical or its intermediates, thereby reducing the overall oxidation efficiency. This is consistent with the observation that PDA-2, treated in the presence of methanol, has a lower quinone content. An alternative possibility is that methanol alters the swelling behavior of PDA or the solubility of the oxidant, leading to a different reaction pathway that favors surface-limited rather than bulk oxidation. While the exact mechanism requires further investigation, the key finding is that the solvent composition provides a simple handle to tune the oxidation level and, consequently, the optical properties of PDA. PDA-1 with higher quinone content exhibits enhanced UV absorption (300–450 nm) and reduced visible–NIR absorbance, whereas PDA-2 with moderate quinone content shows a uniformly lower absorbance across the entire spectrum, resembling a scaled-down version of PDA-0.

### 2.5. Mass Spectrometric Analysis

**Figure 7:**
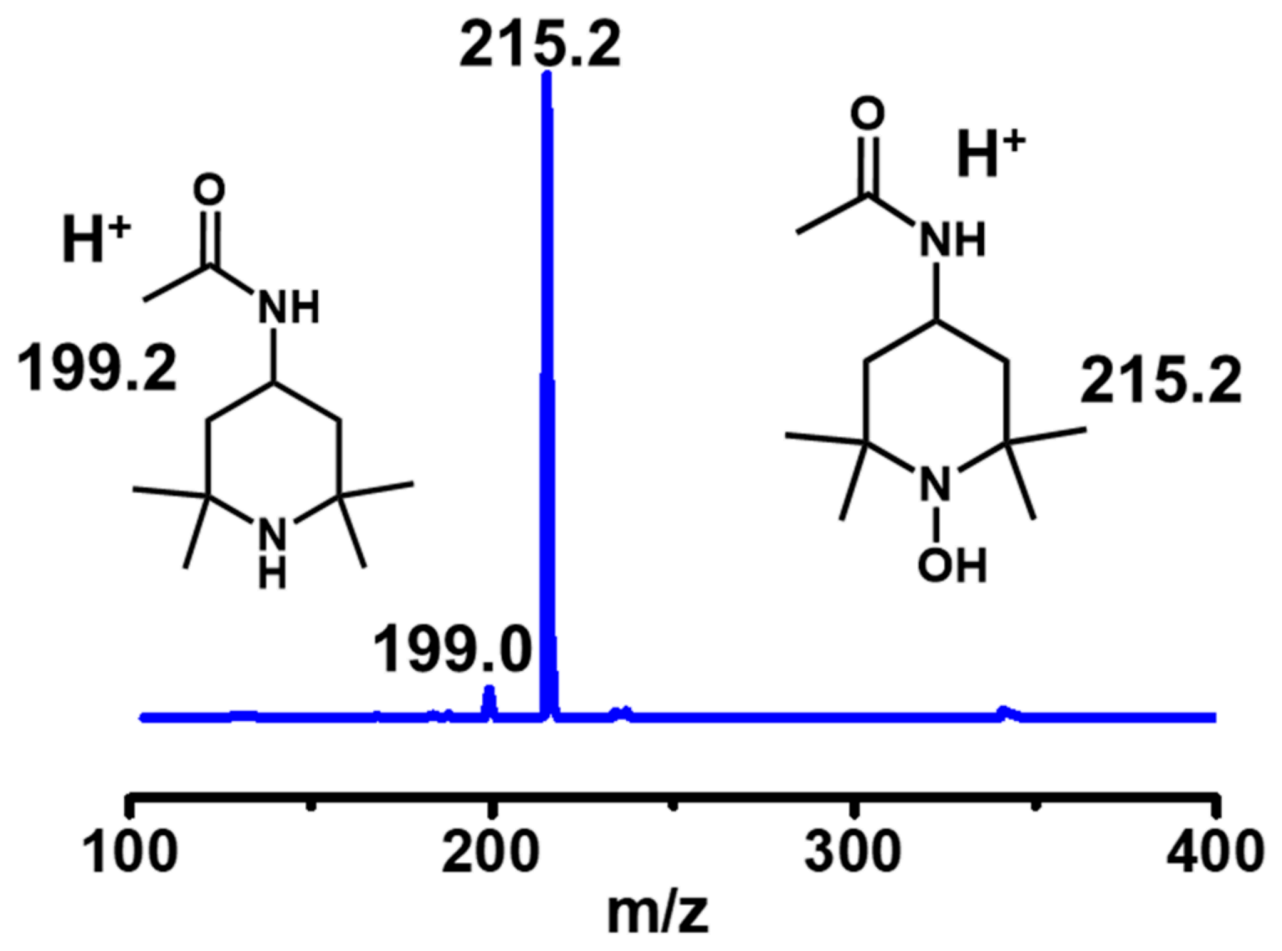
MS spectra of the reaction mixture.

Mass spectrometry was employed to analyze the reaction mixture after the treatment. Two distinct peaks were observed at *m*/*z* 199.0 and 215.2, which correspond to 2,6-di-tert-butyl-4-acetamidopiperidine and 2,6-di-tert-butyl-4-acetamido-1-hydroxypiperidine, respectively (Figure 7). The parent nitroxide radical is not directly detectable under ESI positive conditions, because it rapidly undergoes hydrogen atom abstraction from the protic solvent or environment to form the corresponding N-OH species, which is then readily protonated to generate the observed [M + H]^+^ ion. The signal at *m*/*z* 215.2 corresponds to the [M + H]^+^ ion of 2,6-di-tert-butyl-4-acetamido-1-hydroxypiperidine, the reduced N-OH form of Bobbit’s salt produced by hydrogen abstraction from the protic solvent during ESI. It is well documented that nitroxide radicals are not directly detectable under ESI positive conditions and instead give rise to such reduced, protonated species [35,36]. The peak at *m*/*z* 199.0 is assigned to another reduced fragment. The presence of these reduced forms confirms that Bobbit’s salt acted as an oxidizing agent and was itself reduced during the reaction with PDA. No other peaks were detected in the mass range from 100 to 400, indicating that no soluble polydopamine fragments or oligomers were released into the solution. This result supports the conclusion that the oxidation reaction occurred on the solid polydopamine nanoparticles without causing substantial polymer degradation or dissolution. Consequently, the structural integrity of the polydopamine particles was maintained throughout the chemical modification.

Taken together, the XPS and mass spectrometry data demonstrate that Bobbit’s salt selectively oxidizes the catechol units of polydopamine to quinones while preserving the nitrogen functionality and the overall particle framework. The oxidative transformation is accompanied by the reduction of Bobbit’s salt to its corresponding piperidine derivatives. These findings provide a solid chemical basis for the observed spectral changes and further highlight the potential of Bobbit’s salt as a mild and effective post-synthetic reagent for tuning the electronic properties of polydopamine-based materials.

### 2.6. Optical Properties of Polydopamine-Coated Films

**Figure 8:**
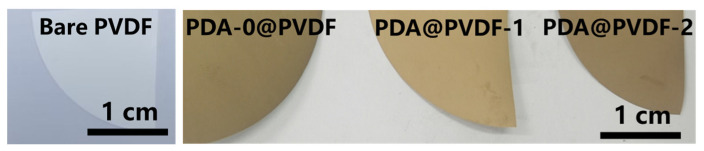
The photographs of bare PVDF, PDA-0@PVDF, PDA@PVDF-1, and PDA@PVDF-2.

**Figure 9:**
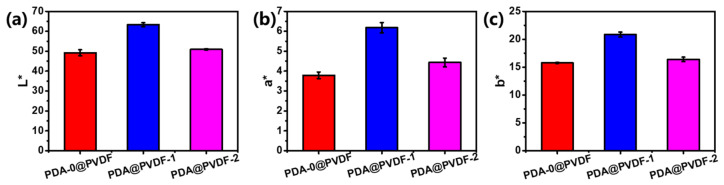
Optical property characterization of different samples: (**a**) L* values, (**b**) a* values, and (**c**) b* values measured using a colorimeter for PDA-0@PVDF, PDA@PVDF-1, and PDA@PVDF-2.

Polydopamine-coated films were prepared by depositing PDA-0 onto PVDF membranes, followed by either no further treatment or immersion in pure water or a 1:1 methanol–water mixture containing Bobbit’s salt. After the immersion, the membranes were gently rinsed with deionized water; the rinse solution remained completely clear, indicating that no detectable amount of the PDA coating was washed away. This is consistent with the well-known strong adhesion of PDA to various substrates [37]. The resulting samples were designated as bare PVDF, PDA-0@PVDF, PDA@PVDF-1, and PDA@PVDF-2, respectively (Figure 8). Colorimetric measurements were performed using a blackness meter, and the results are presented in Figure 9. In terms of lightness L* (a coordinate in the CIE La*b* color space ranging from 0 for black to 100 for white, calculated from the reflectance spectrum according to standard method ASTM D2244) [38], PDA-0@PVDF exhibited the lowest value of approximately 48, indicating the darkest appearance (Figure 9a). PDA@PVDF-1 showed the highest lightness at about 61, meaning the brightest color, while PDA@PVDF-2 fell in between with a value of 50. For the red-green channel a, PDA@PVDF-1 displayed the most pronounced red tone with a value around 6.2, and the other two samples showed progressively weaker red contributions (Figure 9b). Similarly, for the yellow-blue channel b, PDA@PVDF-1 gave the highest value near 21, indicating the strongest yellow tone (Figure 9c). For comparison, the bare PVDF membrane was also measured (L ≈ 88, a ≈ −0.5, b* ≈ −2). These colorimetric changes suggest that the Bobbit’s salt treatment, particularly in aqueous solution, effectively lightens the polydopamine coating and shifts its hue toward red and yellow. The conventional PDA-0 coating, in contrast, appears darker and less saturated. This trend is consistent with the earlier observation that PDA-1 possesses a higher quinone content, which likely alters the light scattering and absorption properties of the film.

### 2.7. Optical Performance on PMMA Substrates

**Figure 10:**
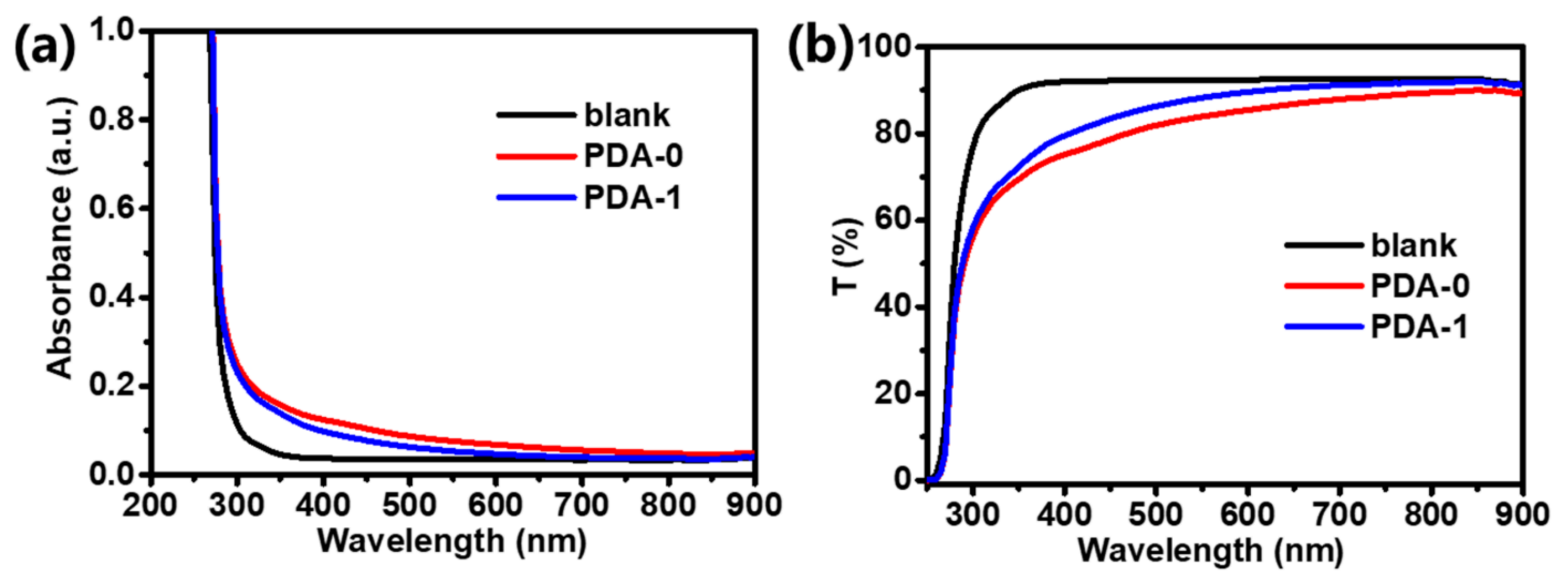
(**a**) UV-Vis absorption spectra and (**b**) transmittance spectra of blank, PDA-0, and PDA-1 samples as a function of wavelength.

To further evaluate the optical performance of the modified polydopamine, PDA-0 and PDA-1 were separately coated onto transparent PMMA substrates. Ultraviolet–visible absorption spectra and transmittance curves were recorded and compared with a blank PMMA sample. As shown in Figure 10, all PMMA-supported samples exhibited a strong absorption peak at approximately 290 nm. The PDA-1-coated PMMA showed the highest absorbance at this wavelength, followed by PDA-0-coated PMMA, while the blank substrate gave the weakest absorption. This indicates that polydopamine coating significantly enhances the ultraviolet blocking capability, and the Bobbit’s salt-treated PDA-1 is even more effective in this regard. The transmittance spectra revealed that the blank PMMA had the highest overall transparency. PDA-0-coated PMMA showed a moderate decrease in transmittance, and PDA-1-coated PMMA exhibited the lowest transmittance across the entire visible range from 400 to 800 nm. The gradual reduction in transmittance correlates with the increased polydopamine loading and the structural modifications induced by Bobbit’s salt. These results clearly demonstrate that the introduction of polydopamine, especially after oxidation with Bobbit’s salt, imparts a strong light shielding effect to the transparent polymer substrate. The combination of enhanced ultraviolet absorption and reduced visible transmittance makes these composite films promising for applications requiring controlled light transmission, such as protective coatings or solar modulating materials.

## 3. Materials and Methods

### 3.1. Materials

Dopamine hydrochloride (98%), ammonium hydroxide solution (25–28 wt%), and methanol (AR, 99%) were purchased from commercial sources and used without further purification. Bobbit’s salt (4-acetylamino-2,2,6,6-tetramethylpiperidine-1-oxoammonium tetrafluoroborate, 95%) was obtained from a chemical supplier. Polyvinylidene fluoride (PVDF) membranes (0.22 μm pore size) were purchased from a local supplier. Poly(methyl methacrylate) (PMMA) sheets (1 mm thickness, optical grade) were used as transparent substrates. All chemicals and materials were purchased from standard commercial suppliers (Sigma-Aldrich, St. Louis, MO, USA) and used without further purification. Deionized water was used throughout all experiments.

### 3.2. Synthesis of PDA-0

Polydopamine nanoparticles were synthesized following a previously reported method with slight modifications [39]. Typically, dopamine hydrochloride (200 mg, 1.06 mmol) was dissolved in a mixture of deionized water (80 mL) and methanol (20 mL) under magnetic stirring at room temperature. After complete dissolution, ammonium hydroxide solution (2.5 mL, 28–30 wt% NH_3_) was added rapidly to the solution. The mixture immediately turned pale brown and then gradually changed to black. The reaction was allowed to proceed for 16 h under open air at room temperature with continuous stirring. The resulting PDA nanoparticles were collected by centrifugation (12,000 rpm, 20 min), washed three times with deionized water, and finally redispersed in water or dried under vacuum at 40 °C for further characterization.

### 3.3. Bobbit’s Salt Treatment of PDA Nanoparticles

The as-synthesized PDA nanoparticles (PDA-0) were subjected to post-synthetic oxidation using Bobbit’s salt under two different solvent conditions. For the aqueous treatment (PDA-1), PDA-0 (25 mg) was dispersed in a Bobbit’s salt solution (200 mg in 10 mL deionized water) giving a mass ratio of 1:8. The mass ratio of 1:8 (PDA to Bobbit’s salt) was chosen after preliminary screening, as it gave the most pronounced spectral changes; further increasing the ratio did not lead to additional improvement. The dispersion was stirred at room temperature for 12 h. For the mixed-solvent treatment (PDA-2), PDA-0 (25 mg) was dispersed in a solution of Bobbit’s salt (200 mg) in a 1:1 (*v*/*v*) methanol–water mixture (10 mL) and stirred under the same conditions. After the reaction, the treated nanoparticles were collected by centrifugation, washed three times with deionized water to remove residual Bobbit’s salt and byproducts, and then dried or redispersed as needed.

### 3.4. Preparation of PDA-Coated PVDF Membranes

PVDF membranes were cut into quarter circle pieces and cleaned by ultrasonication in ethanol for 10 min, followed by deionized water rinsing and drying. For the untreated control, a PDA-0 dispersion (1 mg·mL^−1^ in water, 2 mL) was deposited onto the membrane by vacuum filtration, followed by drying at 60 °C for 30 min to obtain PDA-0@PVDF. For Bobbit’s salt-treated coatings, the PDA-0-coated membranes were then immersed either in pure water containing Bobbit’s salt (20 mg·mL^−1^) or in a 1:1 methanol–water mixture containing Bobbit’s salt (20 mg·mL^−1^) for 12 h at room temperature. The resulting membranes were thoroughly washed with water and dried, yielding PDA-1@PVDF-1 and PDA-1@PVDF-2, respectively.

### 3.5. Preparation of PDA-Coated PMMA Substrates

PMMA sheets (2 cm × 2 cm) were cleaned by ultrasonication in ethanol and deionized water sequentially, then dried. A PDA dispersion (1 mg·mL^−1^ in water, 0.5 mL) was spin-coated onto the PMMA surface at 1000 rpm for 30 s. The coated samples were then immersed in Bobbit’s salt solution (20 mg·mL^−1^ in water) for 12 h at room temperature to obtain the modified coating. After treatment, the samples were rinsed with water and dried. Uncoated PMMA was used as a blank reference.

### 3.6. Characterization

The morphology and particle size of PDA nanoparticles were examined by scanning electron microscopy (SEM, ZEISS GeminiSEM 300, Oberkochen, Germany) operated at an accelerating voltage of 5 kV. Average particle diameters were determined by measuring at least 100 particles from multiple SEM images using ImageJ 1.54p software. Ultraviolet–visible–near-infrared (UV-vis-NIR) absorption spectra of nanoparticle dispersions (0.1 mg·mL^−1^ in water) and coated films were recorded on a UV-vis-NIR spectrophotometer (Shimadzu UV-2600 spectrometer, Duisburg, Germany) over the wavelength range of 300–900 nm. Transmittance spectra of PMMA-supported coatings were measured in the same wavelength range. Fourier transform infrared (FTIR) spectra were collected on an FTIR spectrometer (IS50, Thermo Fisher, Waltham, MA, USA) using the attenuated total reflectance (ATR) mode over the range of 4000–500 cm^−1^ with a resolution of 4 cm^−1^. All spectra were baseline-corrected. X-ray photoelectron spectroscopy (XPS) measurements were performed on an XPS spectrometer (Scientific K-Alpha, Thermo Fisher, Waltham, MA, USA). Survey scans were acquired at a pass energy of 100 eV, and high-resolution O 1s and N 1s spectra were recorded. Binding energies were calibrated using the C 1s peak at 284.8 eV. Peak deconvolution was carried out using commercial software (XPSPEAK 4.1). Mass spectrometry analysis of the reaction supernatant after Bobbit’s salt treatment was performed on a mass spectrometer (Thermo Fisher Scientific, Waltham, MA, USA) equipped with an electrospray ionization (ESI) source in positive ion mode. Colorimetric measurements of the PVDF-supported coatings were conducted using a colorimeter calibrated with a standard white tile. The CIE L*a*b* color coordinates were recorded for three different spots on each sample, and the average values were reported.

## 4. Conclusions

In summary, we developed a simple and effective post-synthetic strategy to tune the optical properties of polydopamine using Bobbit’s salt as a mild and selective oxidant. By treating pre-formed PDA nanoparticles with Bobbit’s salt in either pure water or a 1:1 methanol–water mixture, we obtained two modified materials with distinctly different absorption behaviors. Spectroscopic and spectrometric analyses confirmed that Bobbit’s salt selectively oxidizes catechol units to ortho-benzoquinone moieties while leaving the nitrogen-containing functionalities intact, as evidenced by a marked decrease in the C–O/C=O ratio from 71:29 to 51:49. The extent of oxidation and the resulting spectral changes are solvent-dependent, with the water-treated sample showing generally lower visible–NIR absorbance and the mixed-solvent-treated sample exhibiting enhanced UV absorption but reduced NIR absorption. When applied as coatings on PVDF membranes, the modified PDA produced a brighter, more reddish-yellow appearance compared to the dark, conventional PDA. On transparent PMMA substrates, the modified coating also demonstrated improved UV-blocking capability and reduced visible transmittance. Collectively, these results establish Bobbit’s salt as a convenient and selective reagent for post-synthetic modulation of PDA’s light absorption and color properties. This approach opens new opportunities for designing PDA-based materials for applications in protective coatings, optical filters, and light-management devices.

## Data Availability

The original contributions presented in this study are included in the article. Further inquiries can be directed to the corresponding authors.
